# Human spinal enthesis comparative biology of IL-17F and IL-17A reveals greater T-cell IL-17F induction and IL-23 regulation

**DOI:** 10.3389/fimmu.2025.1658325

**Published:** 2025-11-14

**Authors:** Nicole McDermott, Ala Altaie, Tom Macleod, Charlie Bridgewood, Peter Loughenbury, Robert Dunsmuir, Almas Khan, Vishal Borse, Abhay Rao, Avneet Manghera, Stevan Shaw, Dennis McGonagle

**Affiliations:** 1 Leeds Institute of Rheumatic and Musculoskeletal Medicine (LIRMM), University of Leeds, Leeds, United Kingdom; 2 Leeds Teaching Hospitals NHS Trust, Leeds, United Kingdom; 3 UCB Pharma, Slough, United Kingdom; 4 National Institute for Health Research (NIHR) Leeds Biomedical Research Centre (BRC), Leeds Teaching Hospitals, Leeds, United Kingdom

**Keywords:** spondyloarthritis, IL-17A, IL-17F, cyTOF, enthesis

## Abstract

**Objective:**

Although IL-17F is important in psoriasis, the rudimentary biology of IL-17F in the human enthesis remains undefined. We aimed to characterise IL-23-dependent and independent IL-17F production from entheseal innate and adaptive T cells and determine the impact of IL-17F on entheseal stromal function and mesenchymal stem cell (MSC) osteogenesis.

**Methods:**

Anti-CD3 and anti-CD28 activated human spinal entheseal T cells were immunophenotyped using multi-parameter flow cytometry and a 36-marker Cytometry by Time-Of-Flight (CyTOF) with cytokine profiling, including IL-17A, IL-17F, and TNF (n = 10). IL-17A, IL-17F, and TNF stimulation of entheseal MSC stromal function was evaluated using RNA-seq and by measuring CCL20 protein expression following stimulation with TNF in combination with IL-17A or IL-17F. The osteogenic effects of IL-17A and IL-17F on MSC differentiation were assessed.

**Results:**

Inducible IL-17A and IL-17F expression was predominantly from CD4 T cells and CD4+CD25+ T cells, with higher levels of IL-17F at 72 hr. IL-23 significantly increased IL-17F (p ≤ 0.0001) but not IL-17A. Either IL-17A or IL-17F in combination with TNF dramatically upregulated CCL20 protein expression and substantially changed entheseal stromal transcriptome, with differences in gene expression and pathway activation seen between IL-17A or IL-17F stimulation. However, entheseal MSC osteogenesis was not significantly changed.

**Conclusions:**

There was differential induction of IL-17A and IL-17F from innate and adaptive entheseal T cells, with further significant IL-17F augmentation by IL-23, but not IL-17A. Furthermore, the synergistic effects of IL-17A or IL-17F and TNF on stromal function provide the basis for further enthesis IL-17F biology interrogation.

## Introduction

Seronegative spondyloarthropathies (SpAs) encompass axial and peripheral inflammation, including axial spondylitis (axSpA) and psoriatic arthritis (PsA), and are immunogenetically, experimentally, and therapeutically linked to the IL-23/IL-17 axis. Particularly, IL-17 pathway blockade, but not IL-23 cytokine blockade, has been shown to impact the entire spectrum of osteoarticular pathology; however, IL-17 inhibition has been linked to flares in inflammatory bowel disease (IBD) (1–3). Although there are six members of the IL-17 family, most of the research to date has focused on IL-17A, given the recently available IL-17A targeting monoclonal antibodies, including secukinumab and ixekizumab. In addition to targeting IL-17A, newly available simultaneous targeting of IL-17F appears to show enhanced efficacy compared to inhibition of IL-17A alone in psoriasis (4).

IL-17F is a pro-inflammatory cytokine with 50% homology to IL-17A, with both cytokines being linked to the pathobiology of SpA and experimentally induced bowel inflammation (5). IL-17A and IL-17F can exist as homodimers or heterodimers, all of which are able to bind the IL-17RA and IL-17RC receptor complex (6). Both IL-17A and IL-17F regulate very similar sets of genes, albeit IL-17A appears to induce higher levels of inflammation (7). The main gene pathways activated are the NF-κB pathway and the MAPK pathway, leading to the upregulation of inflammatory cytokines such as IL-6 and members of the CSF family (8–11). IL-17A and IL-17F recruit neutrophils to sites of inflammation via the induction of chemokines such as CXCL2 and IL-8. Both IL-17A and IL-17F have been shown to act on bone marrow-derived and periosteal mesenchymal stem cells (MSCs) and promote osteogenic repair, a potentially highly relevant translational observation given that new bone formation in axSpA occurs in the post-inflammation phase of SpA disease (12, 13).

IL-17A, IL-17F, and IL-22 are important cytokines in barrier protection and are produced by various innate and adaptive T cells, including Th17 and Tc17 cells, which are characterised by the expression of transcription factor RORγt (14–17). Compared to the skin and intestinal epithelial boundaries, which are exposed to microbiota antigenic challenges, the joint (including the enthesis) was thought to be sterile, yet it contains innate and adaptive T cells with inducible IL-17A. The overproduction of IL-17 cytokines has been linked to excessive post-inflammation new bone formation and syndesmophytes at entheseal locations (6, 18).

Whilst the head-to-head data in psoriasis has established superior efficacy of dual IL-17A and IL-17F in the skin compared to other biologic disease modifying anti-rheumatic drugs (bDMARDS) such as adalimumab and secukinumab, similar efficacy was observed at the joint compared to anti-TNF. Due to the lack of head-to-head clinical trials in PsA and axSpA, the sitiuation in the joint is less clear (19–21). However, trials focusing on the joint have found that in TNF failures in PsA and axSpA, the efficacy of bimekizumab remains remarkably high, comparable to that in bio-naïve patients as reported in the BE COMPLETE (22, 23), BE MOBILE, and BE OPTIMAL studies (19, 24). Whilst knowledge has accrued on IL-17A biology with the success of IL-17A monoclonal antibodies, the biology of IL-17F in the normal human enthesis, the cardinal lesion in the SpA spectrum pathology, is rudimentary.

Although reverse translational immunology from clinical trials shows the involvement of both IL-17A and IL-17F in psoriasis, the situation in the skeleton is less clear. Furthermore, IL-17A and IL-17A/IL-17F dual blockade are successful in axial disease (24, 25), yet IL-23 blockade, which acts upstream of IL-17A and IL-17F, was ineffective in axSpA. This raises the possibility that IL-23 inhibition of IL-17A and IL-17F results in different impacts compared to the direct inhibition of IL-17A and IL-17F. Additionally, there are no data on the potential additive or synergistic activity of pivotal cytokines, including IL-17A, IL-17F, and TNF, in the enthesis, and thus, there is a lack of potential immunological insights into optimal SpA therapy developments.

In this work, we investigated the biology of IL-17F in the human spinal enthesis using an *in vitro* model system whereby normal spinous process immune cell populations were investigated, with a specific focus on innate and adaptive T-cell populations, which are known producers of IL-17F at other locations, including the skin. We also looked into the kinetics and magnitude of the entheseal lymphocyte production of both IL-17A and IL-17F. We also determined the downstream effects of IL-17A and IL-17F on stromal function and MSC osteogenesis from spinal peri-entheseal bone and soft tissue to better define potential immune-enthesis stromal crosstalk.

## Methods

### Mechanical isolation of immune cells

Human spinous processes from spinal decompression surgeries were obtained from Leeds Teaching Hospitals using previously described UK research ethics (REC:16/NW/0797) (26). Entheseal samples (n = 25) were used for stimulation assays for cytokine analysis and immunoprofiling by flow cytometry (n = 5), CyTOF (n = 5), ELISA (n = 3), or LEGENDplex (n = 4), and for MSC assays (n = 8). Entheseal soft tissue was separated from the peri-entheseal bone (PEB), and the immune cells were flushed from the bone using phosphate buffered saline (PBS) washes. Soft tissue was digested with collagenase for 3.5 hr with rotation at 37.5°C (n = 5), washout from bone and soft tissue were filtered through a 70um filter, and red cell lysis was performed. Matched blood was also collected, whereby red cell lysis was performed prior to stimulations for flow cytometry and CyTOF analysis (n = 10).

### T-cell stimulations

Following mechanical isolation, the immune cell population was seeded at 1 × 10^6^ cells/mL, and 200,000 cells were added per well in 96-well plates. The 96-well plates were coated with anti-CD3 (100 ng/mL), and soluble anti-CD28 (100 ng/mL) was added to the culture media Roswell Park Memorial Institute (RPMI). T cells were activated for over 72 hr unless stated otherwise, and cytokine production was assessed using either flow cytometry or CyTOF. To assess IL-17A and IL-17F expression, soluble IL-23 (50 ng/mL) was added to the stimulation conditions. Supernatants were collected at 5, 24, 48, and 72 hr, and cells were seeded at a higher concentration of 2.5 × 10^6^/mL. IL-17A and IL-17F levels were measured using specific ELISA kits following the manufacturer’s instructions (Life Technologies, Paisley, PA4 9RF, UK).

IL-23 inhibition was performed using a p19 neutralising antibody (Invitrogen, Paisley, PA4 9RF, UK), and cells seeded at 1 × 10^6^/mL were cultured for 72 hr with no stimulation or combined with anti-CD3/anti-CD28 (100 ng/mL), anti-CD3/anti-CD28^+^IL-23 (50 ng/mL), anti-CD3/anti-CD28^+^IL-23^+^p19i (20 ng/mL), or IL-23 alone (50 ng/mL). Supernatants were collected, and cytokine levels were measured using LEGENDplex.

### Flow cytometry

Intracellular flow cytometry was performed for the assessment of IL-17A and IL-17F; subsequently, GolgiPlug (1:500) was added for 3 hr prior to staining. Viability staining was performed using Zombie NIR fixable dye (BioLegend, London, NW5 1LB, UK), and cells were blocked for 10 min with a human-IgG blocking buffer (**Table 1**). A T-cell panel was used for surface staining (CD45-RF710, CD3-BUV395, CD4-FITC, CD8-PB, CD161-BV510, and Vα7.2-BV756). Cells were fixed and permeabilised using BD Cytofix/Cytoperm Fixation/Permeabilization kit, and intracellular staining was performed for cytokine expression (IL-17A-APC, IL-17F-PE, and TNFα-CFPE594).

**Table 1:** Flow cytometry panel.

Live/Dead	Zombie NIR
CD45	RF700
CD3	BUV395
CD4	FITC
CD8	PacificBlue
CD161	BV510
Vα7.2	BV786
IL-17F	PE
IL-17A	APC
TNFα	CF-PE594

Surface staining of the MSCs to phenotype before the osteogenic differentiation was performed using the following panel: Viability-7AAD, CD45-V450, CD34-APC, CD73-BV421, CD90-PE, and CD105-FITC.

### CyTOF barcoding and staining

Following optimisation experiments using conventional flow cytometry, immune cells from the peri-entheseal bone and peripheral blood were mechanically isolated and cultured unstimulated or stimulated with anti-CD3 and anti-CD28 for 72 hr. Three hours before the end of the culture, GolgiPlug was added (1:500), and for the last 15 min, ^103^Rh (1:100) was added to stain for dead cells. Afterward, culture cells were fixed for 5 min in 1.6% paraformaldehyde and frozen at −80°C.

Cells were thawed and resuspended in 10 mL media plus benzonase (25 U/mL), and then the cells were washed twice and counted. Cells were washed twice in PBS/Ethylenediaminetetraacetic acid (EDTA), pelleted, and resuspended in 800 µL of Standard BioTools barcode permeabilisation buffer. Barcodes were thawed and resuspended in 100 µL perm buffer, added to the samples, and incubated for 30 min at Room temperature (RT). The staining master mix was prepared using 2 µL of the self-conjugated antibodies and 1 µL of the Maxpar antibodies (see **Table 2**). Samples were pooled into four groups under unstimulated and stimulated conditions for the peripheral blood whole blood (WB) and the matched PEB, and the master mix was added for 1–2 hr at RT. Samples were washed twice in permeabilisation buffer, resuspended in 4C BD Perm III (methanol), and incubated for 20 min at RT. Samples were washed in Maxpar buffer, resuspended in 4% formaldehyde plus DNA intercalator, and incubated overnight at 4°C. Cells were frozen in dimethyl sulfoxide (DMSO) (10%) and fetal calf serum (FCS) overnight and thawed just prior to undergoing CyTOF. Prior to running samples through the CyTOF machine, cells were washed twice in PBS and resuspended in cell acquisition solution (CAS) solution at 1 × 10^6^ cells/mL with the addition of EQ beads (1:5).

**Table 2:** CyTOF panel.

Target and clone	Conjugate
CD45 (HI30)	89Y
CD271 (ME20.4)	141Pr
CD19 (HIB19)	142Nd
CD123 (6H6)	143Nd
IL-4 (MP4-25D2)	144Nd
CD4 (RPA-T4)	145Nd
CD8a (RPA-T8)	146Nd
IL-6 (MQ2-13A5)	147Sm
CD16 (3G8)	148Nd
CD69 (FN50)	149Sm
IL-22 (22URTI)	150Nd
CD31 Clone: WM59	151Eu
CD25 (M-A251)	152Sm
TCR VA 7.2 (3C10)	153Eu
CD11c (3.9)	154Sm
TNF (Mab1)	155Gd
IL-17A (eBio64CAP17)	156Gd
IL-10 (JES3-9D7)	158Gd
GM-CSF (BVD2-21C11)	159Tb
CD14 (61D3)	160Gd
CD161 (DX12)	161Dy
CD66b	162DY
IFNy (45–15)	163Dy
TCRγδ (B1)	164Dy
LLT1	165Ho
CD34 (581)	166Er
CD197/CCR7 (G043H7)	167Er
CD45RA (HI100)	169Tm
CD3 (UCHT1)	170Er
CD45RO (UCHL1)	171Yb
IL-17F (SHLR17)	172YB
CD56/NCAM (RDR5)	173YB
HLA-DR (L243)	174Yb
CD127 (B159)	175Lu
CD11b (ICRF44)	176Yb

### Cytokine measurement

IL-17A, IL-17F, and CCL20 were measured using ELISA (Invitrogen) following the manufacturer’s protocol. Conditioned media collected after 72 hr of T cell receptor (TCR) activation were measured using a Th17 LEGENDplex assay (BioLegend). The LEGENDplex assay was performed following the manufacturer’s instructions, and measurements were taken using a CytoFLEX S cytometer.

### Entheseal MSC stimulation with stromal function evaluation

Entheseal MSCs were isolated from bone and soft tissue using collagenase digest for 3.5 hr at 37°C. MSCs were cultured and expanded using StemMACs media (Miltenyi, Woking GU24 9DR, UK) until confluency was reached. Cells were seeded at 50,000 cells/mL MSCs and plated in 24-well plates at 1 mL per well. Cells were plated and allowed to adhere for 4 hr, and then the complete medium was replaced with StemMACs supplemented with IL-17A (100 ng/mL), IL-17F (100 ng/mL), and TNF (10 ng/mL). After 48 hr, supernatants were collected to assess MSC activation via CCL20 measurement using ELISA, and the cells were lysed in TRIzol for RNA extraction and gene expression analysis (bulk sequencing).

### RNA bulk-sequencing analysis

Library construction, quality control, and sequencing were performed by Novogene. First- and second-strand syntheses were performed before samples were barcoded with Illumina-compatible adapters. The libraries were pooled and sequenced on Illumina platforms. Raw reads obtained by sequencing were mapped to the hg38 reference genome, with the reference genome and gene model annotation files downloaded directly from a genome database. An index of the reference genome was built using HISAT2 v2.0.5, and paired-end clean reads were aligned to the reference genome using the same tool. Differential gene expression analysis was performed using DESeq2, with pAdj < 0.05 and fold change > ± 2.0 set as the arbitrary cutoff threshold for statistical significance. Volcano plots were constructed for data visualisation in R (V.4.4.1), utilising EnhancedVolcano with FCcutoff = 1 and pCutoff = 0.05.

Gene set enrichment analysis (GSEA) was conducted using the clusterProfiler package in R. Prior to running GSEA, the gene expression data were pre-processed and filtered to generate a ranked list of genes based on their differential expression between experimental conditions. Subsequently, GSEA was performed using the gseGO function in clusterProfiler, which conducts enrichment analysis based on Gene Ontology (GO) terms. The output of GSEA included enrichment scores, p-values, and false discovery rates (FDRs) for each GO term, with GO terms exhibiting significant enrichment scores and low FDR values considered biologically relevant, as well as pathway enrichment analysis.

### Osteogenic differentiation

MSCs were cultured and expanded using StemMACs media (Miltenyi) until confluency was reached. Cells were seeded at 10 × 10^4^ cells/mL in StemMACs media and allowed to attach to the plates for 4 hr before the complete medium was replaced with OsteoDiff media (Miltenyi) with/without cytokines (IL-17A and IL-17F 50 ng/mL) or supplemented with conditioned media 1:7 (supernatants from activated T cells). Half media changes were performed twice a week until day 21. Osteogenesis was measured at day 14 by alkaline phosphatase production and at day 21 by calcium production. Staining and quantification methods for both alkaline phosphatase and calcium are previously described by Russell et al. (6).

### CyTOF analysis

Using the CyTOF software, samples were debarcoded, and FCS files were imported into the FlowJo analysis software. FCS files for the PEB were concatenated, merging all donors under the unstimulated and stimulated conditions for comparison between conditions, giving an overall view of the phenotypic and cytokine profiles. Manual gating was performed to remove EQ beads and doublet cells. Gating of the live cells was performed, and then Uniform Manifold Approximation and Projection (UMAP) and flow self-organizing map (FlowSOM) clustering were performed using all markers (**Table 2**). Manual gating was performed to identify the individual immune cell subsets making up the clusters. These were identified from three main populations—CD45^+^CD3^+^, CD45^+^CD3^−^, and CD45^+^CD3^−^CD19^−^—and the frequency of parent populations was plotted under both the unstimulated and stimulated conditions.

For the T-cell analysis, CD45^+^CD3^+^ cells were manually gated under the stimulated condition, as we wanted to assess cytokine production. UMAP and FlowSOM were performed, and from this clustering, T-cell subsets were identified, as well as cytokine expression.

### Statistical analysis

All statistical analyses were performed using either a one-way or two-way ANOVA, depending on the data set, with Tukey’s multiple comparison test. Statistical significance was reached at a p-value <0.05.

## Results

### Predominant expression of IL-17F over IL-17A in the spinal enthesis

**Figure 1:**
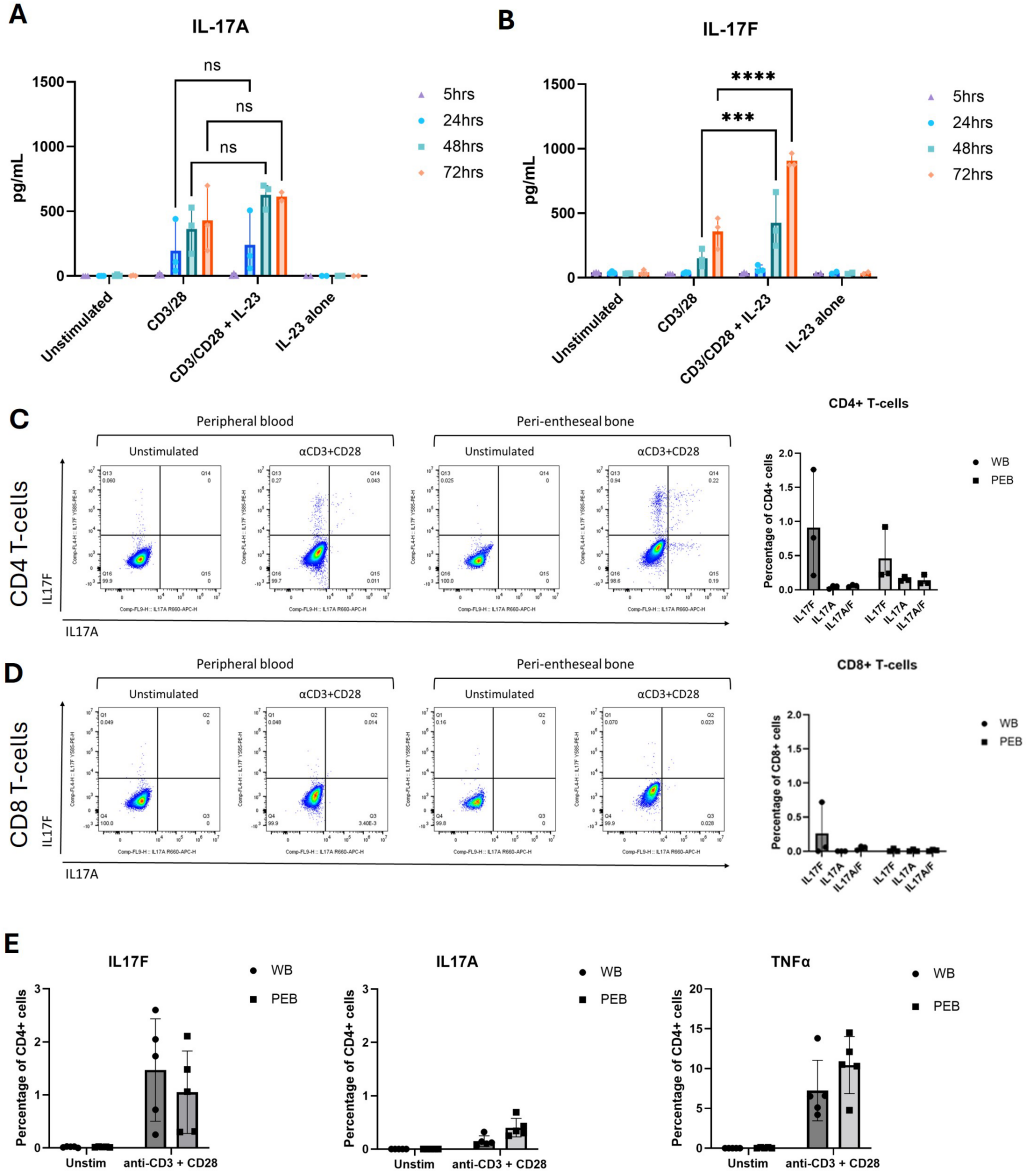
IL-17F is the predominant isoform expressed in the human enthesis. **(A, B)** ELISA of IL-17A and IL-17F secretion from entheseal T cells stimulated with anti-CD3/CD28 (100 ng/mL each) with/without IL-23 (50 ng/mL) over 5–72 hr. Values represent mean ± SD; n = 3 donors; each dot represents an individual donor. **(C, D)** Intracellular flow cytometry of CD4^+^**(C)** and CD8^+^**(D)** T cells showing IL-17A (x-axis) and IL-17F (y-axis) expression after 72-hr stimulation. Percentages of CD4^+^ and CD8^+^ T-cell expression of IL-17F or IL-17A or their co-expression were quantified. **(E)** Comparison of IL-17A, IL-17F, and TNF expression from CD4^+^ T cells in matched peripheral blood (WB) and peri-entheseal bone (PEB). Each dot represents an individual donor (n = 5). Values are mean ± SD. Statistical analysis was performed using two-way ANOVA with Tukey’s multiple comparisons. NS - not significant, *** - 0.0003, **** - <0.0001.

Due to the phenomenon that IL-23 inhibitors do not work for axial SpA, but IL-17A and dual IL-17A/IL-17F inhibitors had successful phase 3 trial data, firstly, the contribution of IL-23 on IL-17A and IL-17F production was investigated. ELISA was used to measure IL-17A and IL-17F from supernatants collected between 5 and 72 hr following TCR activation and the addition of IL-23 to the stimulation condition. This revealed that IL-17A expression (mean = 240.58 pg/mL) was greater than IL-17F expression (mean = 68.85 pg/mL) at 24 hr. However, by 72 hr, there was greater expression of IL-17F (mean = 905.41 pg/mL) than IL-17A (mean = 614.67 pg/mL) (**Figures 1A, B**), in keeping with data previously derived from the skin and blood (27). Secondly, it was noted that the addition of IL-23 to CD3/CD28 activated T cells significantly increased IL-17F (p ≤ 0.0001) production but not IL-17A (**Figures 1A, B**). IL-23 alone did not induce IL-17A or IL-17F, but in the presence of TCR stimulation, it selectively augmented IL-17F production, consistent with an IL-23R-mediated but not IL-23-dependent effect (**Figure 1B**; **Supplementary Figures 1**, **2**).

Along with Th17 cytokines, other inflammatory cytokines were measured including TNF, IL-6, IFN-γ, IL-22, and IL-10 were also measured using LEGENDplex and showed a substantial increase in response to IL-23 in conjunction with CD3/CD28 stimulation. Although the increase in IL-17A and IL-17F expression from the LEGENDplex data did not reach significance, this reflected donor variability in response to stimulation, and we confirmed the trend of greater enhancement of IL-17F compared to IL-17A with IL-23 (**Supplementary Figure 2**). Consistent with this, the blockade of IL-23 using a p19 inhibitor reversed the augmentation of IL-17F, returning levels to those observed with anti-CD3/CD28 stimulation alone (**Supplementary Figure 1**). This confirms that the IL-23 effect on IL-17F is additive and reversible.

Initially, intracellular flow cytometry was used to assess the expression of both IL-17F and IL-17A after 72-hr entheseal TCR stimulation, as we showed in **Figure 1A** that the 72-hr timepoint had the highest levels of IL-17A and IL-17F production.

Flow cytometry identifying the sources of IL-17A and IL-17F demonstrated that the CD4^+^ T-cell population was the main producer of IL-17A and IL-17F (**Figure 1C**) and that little to no expression came from the CD8^+^ T-cell population (**Figure 1D**). These analyses also showed that IL-17F was the predominant isoform being expressed after 72 hr, with a low percentage of CD4^+^ T cells expressing IL-17A (**Figure 1D**). Including IL-23 in the flow assays would have disproportionately amplified IL-17F relative to IL-17A, potentially obscuring the baseline comparison of CD4^+^ versus CD8^+^ T-cell contributions.

We also measured TNFα, another key target in SpA, which was 10-fold higher than IL-17F (**Figure 1E**). Using intracellular flow cytometry, we observed that monospecific IL-17F^+^ populations were predominant, with double the percentage of their parent population compared to IL-17A^+^ and IL-17A^+^/IL-17F^+^ populations (IL-17F^+^ 0.46%, IL-17A^+^ 0.15%, and IL-17A^+^/IL-17F^+^ 0.13%) (**Figures 1C, D**). Using conventional flow cytometry, the rarity of some cell types prevented the identification of all immune cell populations present in our *in vitro* model, so we progressed to CyTOF analysis.

### Immunoprofiling and cytokine profiling of normal human enthesis

**Figure 2:**
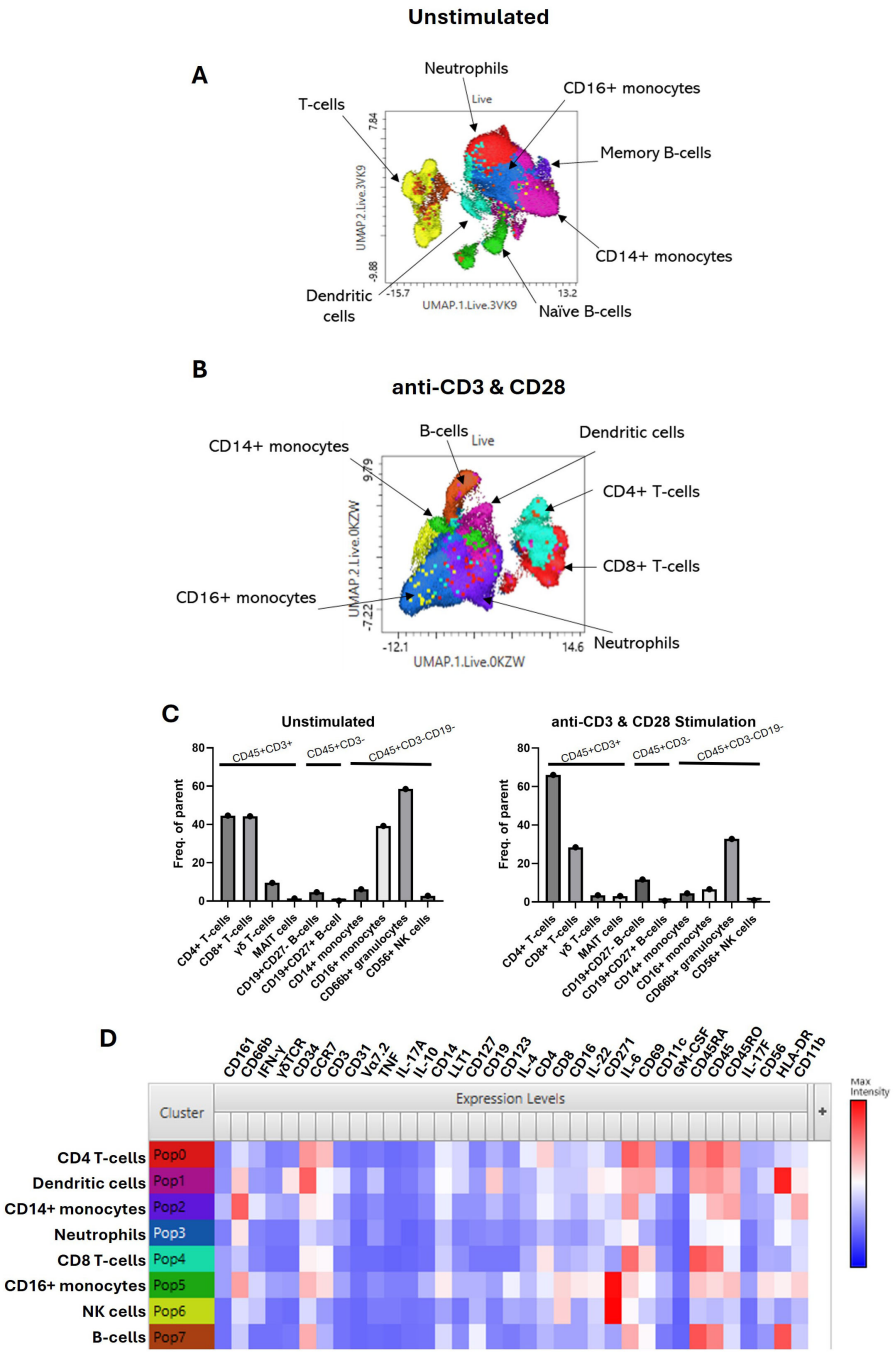
Immunoprofiling of the human enthesis. Entheseal immune cells were cultured for 72 hr with/without TCR activation (anti-CD3 and anti-CD28–100 ng/mL). Cluster analysis was performed on the concatenated data from the five donors to generate UMAP plots from the unstimulated samples **(A)** and the stimulated samples **(B)**. Colours are assigned automatically and, therefore, differ between panels; consequently, annotation was used to identify the cluster. Standard cytometric gating stategies (**Table 3**) were used to further idendify the rarer immune cell populations under the unstimulated and stimulated conditions **(C)**, revealing innate immune cell populations, including the γδ T-cell mucosal-associated invariant T (MAIT) cells and small populations of B cells. Heatmap of marker expression from each identified cluster from the stimulated samples **(D)**, n = 5.

An extensive immune-profiling panel using a 36-marker CyTOF was employed (**Table 2**) for in-depth analysis. Matched peripheral blood and PEB samples from five donors were barcoded, and the concatenated data were analysed by UMAP clustering using FlowJo. Seven main populations were identified from the cluster analysis, FlowSOM, in the unstimulated and stimulated samples (**Figures 2A, B**). Rarer immune cell populations, including the γδ T cells and mucosal-associated invariant T (MAIT) cells, as well as B-cell subsets, were quantified from both the unstimulated and stimulated samples (**Figure 2C**). The three main clusters were identified as CD4^+^ T cells, CD8^+^ T cells, and the CD66b^+^ neutrophil population. TCR stimulation promoted the expansion of CD4^+^ T cells and a reduction in CD16^+^ monocytes. The heatmap indicating the marker expression for each of these clusters (**Figure 2D**) was used to identify each population from the stimulated samples.

**Table 3:** Phenotyping markers.

Cell subset	Markers
CD4^+^ T cells	CD45^+^CD3^+^CD4^+^
CD8^+^ T cells	CD45^+^CD3^+^CD8^+^
γδ T cells	CD45^+^CD3^+^γδTCR^+^
MAIT cells	CD45^+^CD3^+^CD8^+^CD161^+^Vα7.2^+^
CD19^+^CD27− B cells	CD45^+^CD3^−^CD19^+^CD27^−^
CD19^+^CD27^+^ B cells	CD45^+^CD3^−^CD19^+^CD27^+^
CD14^+^ monocytes	CD45^+^CD3^−^CD19^−^CD14^+^CD16^−^
CD16^+^ monocytes	CD45^+^CD3^−^CD19^−^CD14^−^CD16^+^
CD66b granulocytes	CD45^+^CD3^−^CD19^−^CD66b^+^
CD56 monocytes	CD45^+^CD3^−^CD19^−^CD56^+^

MAIT, mucosal-associated invariant T.

### CyTOF cytokine profiling of entheseal T cells

**Figure 3:**
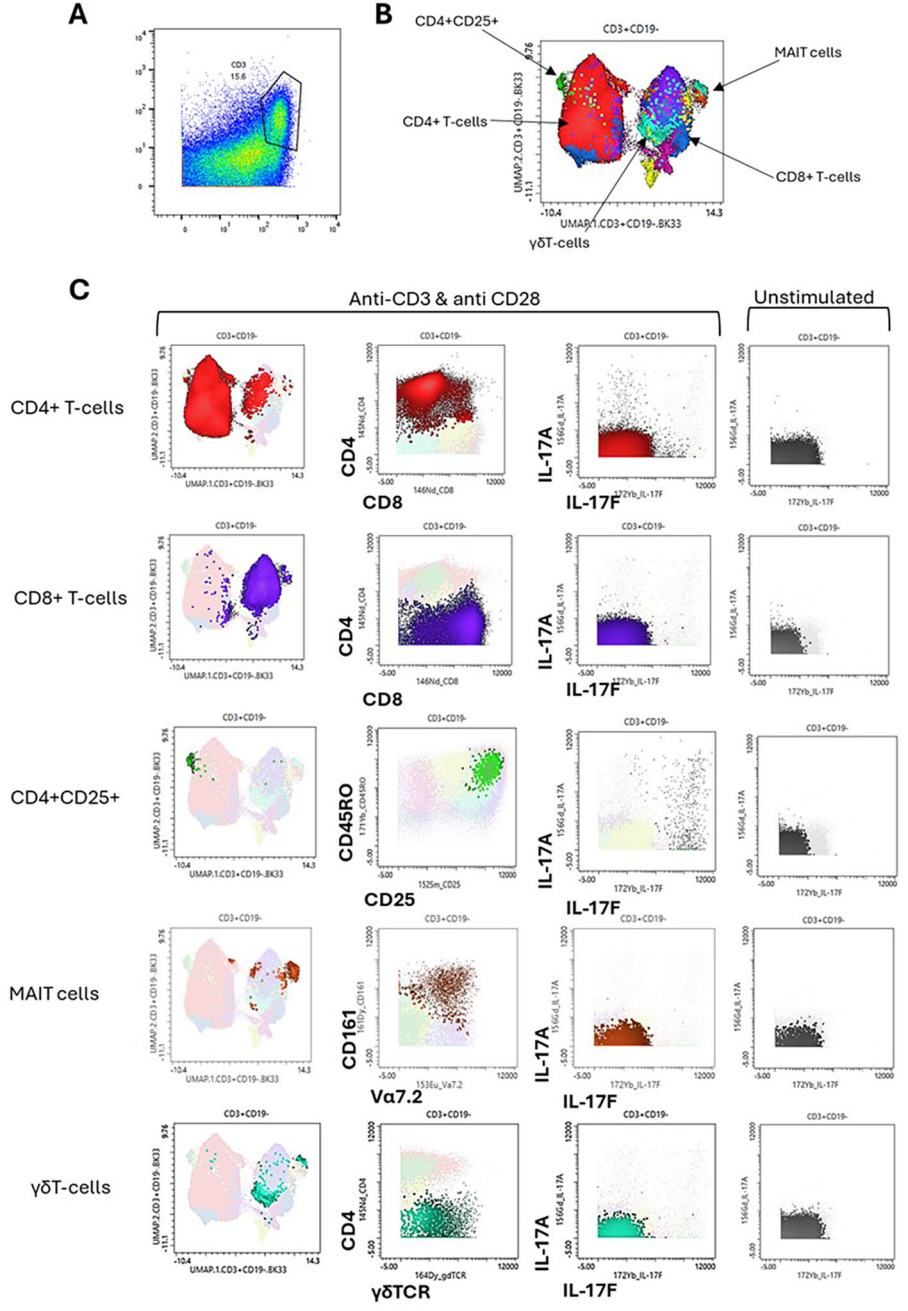
Cytokine profiling of entheseal T cells. Entheseal T cells were assessed for their ability to produce IL-17A and IL-17F. From the stimulated CyTOF data, the T-cell CD3^+^ population was gated **(A)**, UMAP clustering was performed **(B)**, and the phenotype of each cluster was plotted as shown on the x-axis and y-axis **(C)**. IL-17A and IL-17F expression is shown for the main T-cell subsets identified, CD4^+^, CD8^+^, CD4^+^/CD25^+^, mucosal-associated invariant T (MAIT) cells, and γδ T cells **(C)** and compared against IL-17A and IL-17F expression from the same subsets under the unstimulated condition, n = 5.

UMAP clustering from the CD3^+^ gate (**Figure 3A**) showed multiple T-cell subsets present, with the largest cluster being made up of CD4^+^ T cells (**Figure 3B**). The clustering also revealed subsets of CD8^+^ T cells, CD4^+^CD25^+^ T cells, MAIT cells, and γδ T cells (**Figure 3B**). These clusters were phenotyped using phenotypic markers as shown in the second column of the panel (**Figure 3C**). Then, IL-17A and IL-17F production from these clusters was compared, revealing that the majority of expression was seen in CD4^+^ T cells and CD4^+^CD25^+^ T cells, which we confirmed to be CD45RO^+^, indicating an activated memory T-cell subset. Neutrophils, which made up a large proportion of PEB cells, were negative for IL-17F expression under our experimental conditions (**Supplementary Figure 3**).

### Effects of IL-17A and IL-17F on MSC stromal activation

**Figure 4:**
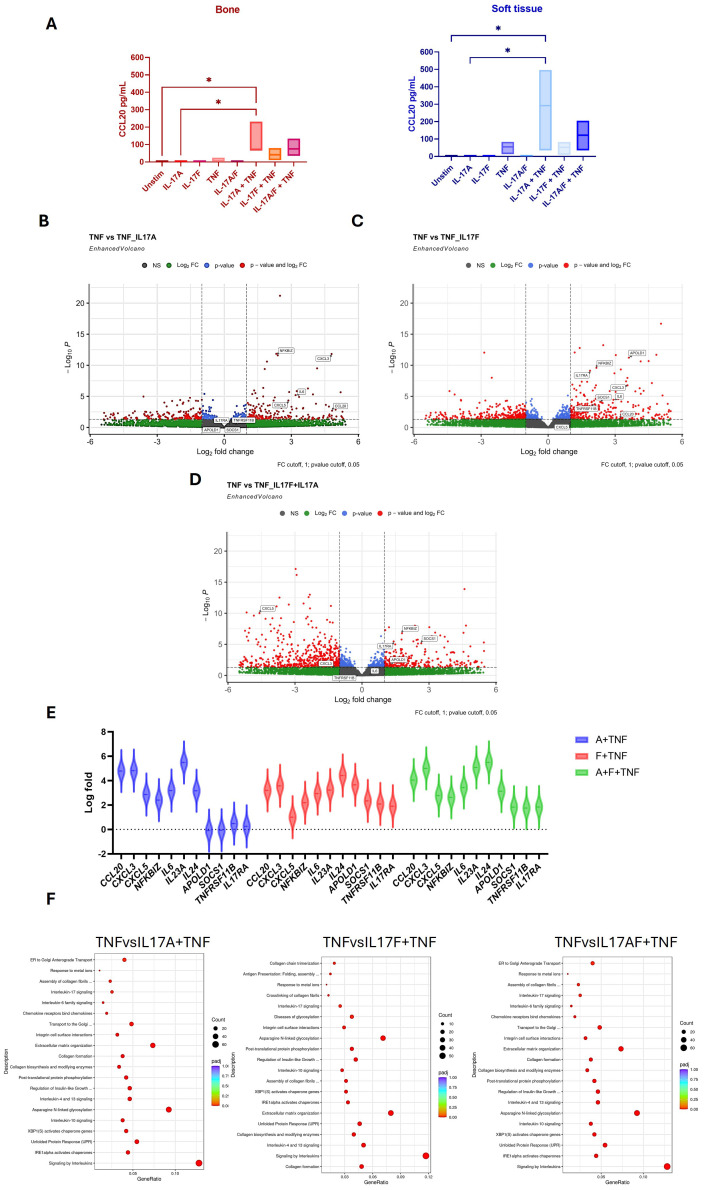
Effects of IL-17A, IL-17F, and TNF on entheseal mesenchymal stem cells (MSCs) isolated from entheseal bone and soft tissue were stimulated with IL-17A (50 ng/mL), IL-17F (50 ng/mL), and TNF alone and in combination for 48 hr, n = 3. Supernatants were collected, and levels of CCL20 were measured using ELISA **(A)**. One-way ANOVA performed with Tukey’s multiple comparison test. RNA collected from the bone marrow-derived MSCs following stimulation was sent for bulk sequencing, and differential gene expression analysis was performed. Differentially expressed genes (DEGs) are displayed as volcano plots between IL-17A+TNF vs. TNF **(B)**, IL-17F+TNF vs. TNF **(C)**, and IL-17A+F+TNF vs. TNF **(D)**. Violin plot showing DEGs up- and downregulated between conditions **(E)**. Pathway enrichment analysis shows the pathways that were upregulated **(F)**. *- <0.03

To investigate potential T-cell and entheseal stromal crosstalk, we evaluated MSC stromal function and activation by measuring CCL20 protein production following stimulation with Th17 cytokines IL-17A, IL-17F, and TNF, as well as using these cytokines in combination (**Figure 4A**). In isolation, these cytokines had relatively little effect on CCL20 production, and the combination of IL-17A and IL-17F showed no additive effect. However, when combining either IL-17A or IL-17F with TNF, CCL20 production was significantly increased in both the bone (p = 0.0273) and soft tissue (p = 0.0197), showing a clear synergistic effect for both IL-17A and IL-17F with TNF and the activation of an inflammatory pathway within the entheseal MSCs. As IL-23 acts upstream to drive IL-17 production, it was not included in the stromal assays, which were designed to examine the direct effector roles of IL-17A and IL-17F (alone or with TNF) on MSC activation.

Differential gene expression (DGE) analysis was utilised to examine transcriptional changes mediated by IL-17A and IL-17F. Given that TNF alone induced baseline CCL20 production (**Figure 4A**), this condition was used as the comparator condition in the DGE analyses (**Figures 4B–D**) for the assessment of the additive and synergistic effects of IL-17A and IL-17F. Comparing the effects of TNF alone to the effects of TNF with IL-17A and IL-17F revealed that there are more upregulated genes when IL-17F and TNF are combined compared to TNF with IL-17A. Volcano plot analysis demonstrated that TNF in combination with IL-17A or IL-17F induced stronger transcriptional responses than TNF alone. The expression of key chemokines such as CCL20, CXCL3, and CXCL5, together with pro-inflammatory mediators including IL-6 and the NF-κB regulator NFKBIZ, was significantly upregulated under the combined conditions. These findings indicate that IL-17A and IL-17F act synergistically with TNF to amplify stromal inflammatory pathways.

The top differentially expressed genes (DEGs) from comparisons made between TNF and TNF with the addition of IL-17A and IL-17F, as well as in combination, are plotted as violin plots (**Figure 4E**). This highlights subtle differences between the effects of IL-17A and IL-17F. Higher expression of *CCL20* and *CXCL3* was seen with the addition of IL-17A and, interestingly, a downregulation of *IL17RA* alongside *APOLD1*, *SOCS1*, and *TNFRSF11B* to a greater extent than with the addition of IL-17F. This may reflect the crosstalk between T cells and MSCs, highlighting the stronger inflammatory role of IL-17A compared to IL-17F, shown by the upregulation of activation genes but also the downregulation of pro-inflammatory/signalling genes such as *IL17RA*, *TNFRSF11B*, and *SOCS1*. This may serve as a feedback loop to regulate inflammation and repair.

Pathway enrichment analysis revealed differences between the pathways upregulated in the combination of IL-17A or IL-17F with TNF. The addition of IL-17A upregulated signalling pathways such as GPCR and IL-4 and IL-13 pathways, whereas IL-17F appears to upregulate pathways involved more in cellular re-organisation, such as extracellular matrix organisation and collagen formation, as well as N-linked glycosylation (**Figure 4F**). Collectively, these findings showed a synergistic effect between IL-17A or IL-17F and TNF on stromal function but subtle differences in stromal inflammatory response at the global transcriptional level.

Finally, unlike previous reports of additive effects of IL-17A and IL-17F on MSC osteogenesis induction in periosteal stem cells, we did not see statistically significant augmentation of osteogenesis with the supplementation of IL-17A and IL-17F. However, a trend for higher degrees of osteogenesis was evident as defined by alkaline phosphatase and calcium levels (**Supplementary Figure 5**).

## Discussion

To the best of our knowledge, the biology of IL-17A and IL-17F has been explored in the skin in psoriasis and to a lesser extent in the synovium in both PsA and RA (28–30), but not previously in the human enthesis. We noted the delayed onset of IL-17F production but higher levels of expression by T cells compared to IL-17A, which could theoretically be relevant for the more chronic phases of entheseal inflammation, but further studies are needed. We also noted that both IL-17A and IL-17F augmented entheseal stromal cell function via CCL20 in a synergistic manner with TNF, which was confirmed at the transcriptional and protein levels. We investigated the effects of IL-23, a cardinal regulator of IL-17A and IL-17F, and found a novel impact of IL-23 on driving greater IL-17F induction from activated T cells compared to IL-17A, and we defined the downstream relationship of IL-17F on entheseal stromal function.

We reveal for the first time the differences between IL-17A and IL-17F entheseal expression profiles, as well as the enhanced expression of IL-17F with IL-23 stimulation. In the enthesis, we observed that CD4^+^ T cells and CD4^+^CD25^+^ are responsible for the majority of IL-17F production (**Figures 1**, **3**), with a small percentage of CD4^+^ T cells co-expressing both IL-17A and IL-17F. Cytometric analysis by CyTOF confirmed that the IL-17F expression comes from the CD4^+^ compartment and also correlates with CD25^+^CD45RO^+^, indicating activated memory T cells. However, in diseases linked to MHC-I genes, including psoriasis, PsA, and axSpA, it is likely that tissue-resident memory (TRM) CD8^+^ T cells are key producers of both IL-17A and IL-17F; future studies should aim to address this (31–33). We have also shown that the addition of IL-23 to stimulation conditions significantly increases the production of IL-17F compared to IL-17A, which infers that IL-17F is more responsive to IL-23 and, therefore, may be important in IL-23-driven disease and perhaps have a greater dependence on its induction compared to IL-17A. Our data indicate that IL-17F expression in the enthesis is primarily TCR-driven but can be further augmented through IL-23R signalling, consistent with a model of IL-23R-mediated rather than IL-23-dependent regulation.

The increased activation of entheseal stroma following concomitant TNF stimulation with both IL-17A and IL-17F, but not when these two cytokines were used together (**Figure 4**), is in keeping with prior reports from RA fibroblasts (7, 27, 28). This highlights the synergistic effects of IL-17A and IL-17F on other inflammatory cytokines, but alone, they are unable to drive stromal activation. The overlapping biology and activation of peripheral tissue by both IL-17A and IL-17F have previously been reported (27). Our focus on CCL20 is compatible with the ability of TNF and either IL-17A or IL-17F to recruit CCR6-positive T cells with a bias towards IL-17A- and IL-17F-producing cells (34, 35). The NF-κB pathway was upregulated in MSCs stimulated with TNFα, which is known to mediate MSCs’ immunosuppressive capability (36–38). The co-stimulation of TNF with IL-17A or IL-17F leads to the significant upregulation of *NFKBIZ*, which encodes IκBζ, an inhibitor of NF-κB signalling (39), thus reducing the immunosuppressive capacity of the MSCs but also inducing inflammation-mediated genes involved in SpA disorders, such as *CCL20* (40). This mechanism of synergistic stromal cell activation that requires TNF does not readily lend an explanation for how TNF inadequate responder (TNF-IR) patients with PsA and axSpA have shown similar efficacy as bio-naïve patients following IL-17A and IL-17F dual blockade, and further work is needed. As IL-23 primarily acts upstream to regulate IL-17 production rather than directly affecting stromal cells, we did not test its effects in the stromal assays. Instead, these experiments were designed to assess the downstream effector roles of IL-17A and IL-17F, particularly in synergy with TNF.

In bio-naïve patients with PsA, the efficacy of dual inhibition of IL-17A and IL-17F on ACR responses in the BE OPTIMAL trial was comparable to that in historical trials with IL-17A or TNF blockers (19). However, the efficacy of the dual blockade of IL-17A and IL-17F in PsA patients with prior TNF failure in the BE COMPLETE trial was comparable to that in bio-naïve patients (22), unlike the general trend in clinical trials. Likewise, a *post-hoc* analysis of bio-naïve versus TNF-IR patients in the BE MOBILE 1 (nr-axSpA) and two (r-axSpA) phase 3 trials suggested that the response rates were similar in patients who were bio-naïve compared to TNF-IR patients (22). Against such an enigmatic backdrop and differences between the skin and the joint in trials, we provide the first rudimentary biology of IL-17F in the enthesis in relation to its sister cytokine IL-17A, demonstrating differences between the two cytokines.

We did not replicate the finding of a synergistic impact of IL-17A and IL-17F on osteogenesis that was previously reported for periosteal stem cells (13). However, the fact that both IL-17A and IL-17F resulted in numerical increases in MSC osteogenesis, especially on spinal soft tissue, is in keeping with prior reports of these two cytokines being pro-osteogenic (6). This raises the question of whether IL-17A and IL-17F dual blockade may have potential in suppressing entheseal new bone formation more than the inhibition of IL-17A alone, the latter of which was not superior to TNF blockers in restraining new bone formation in the spine in humans (41).

Thus far, little is known about what differentiates IL-17A and IL-17F expression levels, but evidence suggests that transcriptional regulation differs between IL-17A and IL-17F, with IL-17A being regulated by NFATc1 and IL-17F by STAT5 (42, 43). Cole et al. (2023) also suggested that the expression of IL-17A or IL-17F or their co-expression was linked to chromatin accessibility and previous antigen exposure (44). Further investigation into the regulatory pathways of IL-17A and IL-17F may be able to account for the IL-23-dependent and independent production of IL-17A and IL-17F. A limitation of our study is that we did not investigate the co-expression of IL-17F with IFN-γ at the single-cell level, which could provide additional insight into the heterogeneity of entheseal T-cell responses.

To summarise, we have reported the first investigation of human enthesis IL-17F biology and show time-dependent differences in IL-17A and IL-17F protein induction from the CD4^+^ T-cell subset, as well as significantly higher IL-17F induction following the addition of IL-23 to activated entheseal T cells. Further work is required to unravel the regulation and signalling cascades responsible for driving these differences. Our findings from normal enthesis immune populations provide a platform to further investigate the basis for why dual IL-17A and IL-17F blockade may have an important role in patients who have inadequately responded to TNF inhibitors. The different transcriptional regulation of IL-17A and IL-17F in such patients requires further investigation in the human enthesis. Future studies are required to validate these findings in murine models of IL-23-driven spondyloarthritis, which would provide additional mechanistic insight and cross-species confirmation of the human data presented here.

## Data Availability

The datasets presented in this study can be found in online repositories. The names of the repository/repositories and accession number(s) can be found in the article/**Supplementary Material**.

## Supplementary material

Supplementary Figure 1 *IL-17F expression from T-cells is enhanced but not dependent on IL-23* Entheseal immune cells were stimulated for 72hrs using different T-cell stimulation conditions as well as IL-23 blockade with a p19 inhibitor. Cells seeded at 1x10^6^/ml were cultured with no stimulation, anti-CD3+anti-CD28 (100ng/ml), anti-CD3+anti-CD28 + IL-23(50ng/ml), anti-CD3+anti-CD28+IL-23+p19i (20ng/ml) or IL-23 alone (50ng/ml). Supernatant was collected after 72hrs and cytokine expression was assessed using a Legendplex assay for multi-analyte detection. N = 4

Supplementary Figure 2 *Fold change of IL-17A and IL-17F expression with the addition of IL23* Fold change calculated between the CD3+CD28 stimulation and CD3+CD28+IL23 stimulation for IL-17A and IL-17F expression, **(A)** fold change is representative of the ELISA data n=3, (B) fold change representative of the Legendplex data n=4. Statistical analysis performed using paired t-test.

Supplementary Figure *IL-17A & IL-17F expression from PEB neutrophils and CD4+ T-cells* CyTOF analysis from PEB samples, cluster analysis performed on the stimulated (anti-CD3 & CD28) to isolate the neutrophil cluster and 2D plot to show IL-17A/IL-17F expression **(A)**. 2D plots of the CD4+ T-cell populations from the unstimulated and stimulated samples showing IL-17A/IL-17F expression **(B).**

Supplementary Figure 5 Heatmaps of the top DEGS from MSCs stimulated with IL-17A, IL-17F and TNF Heatmaps of the up- and down-regulated differentially expressed genes (DEGs) from comparisons between TNF and conditions with the addition of IL-17A and IL-17F. Upregulated genes (red) and down-regulated genes (blue).

Supplementary Figure 5 Effects of IL-17A & IL-17F on osteogenesis. Osteogenic differentiations were set up in OsteoDiff media with the addition of recombinant IL-17A (50ng/ml), IL-17F (50ng/ml) and combined IL-17A/F (25/25ng/ml) and supernatant collected from activated T-cells after 72hr (1:7). StemMACs media was used as a control condition. At days 14 & 21 osteogenesis was assessed via staining methods, lysate quantification as well as RNA collection for gene expression. MSCs isolated from entheseal bone and soft tissue using collagenase digest were culture expanded and phenotyped for MSC markers CD90, CD73 & CD90 **(A)** -bone & **(B)** – soft tissue). Day 14 staining was performed for alkaline phosphatase with duplicate wells and levels of alkaline phosphatase quantified from lysates collected **(C, E)**. Day 21 alizarin red staining performed and calcium quantification **(D, F)**. MSCs isolated from entheseal bone and soft tissue were stimulated for 48hrs in the presence of IL-17A, IL-17F and TNF as well as in combination, MSC activation was measured by CCL20 expression by ELISA **(G)** N = 5
