# Supplementary figures and images for: Human spinal enthesis comparative biology of IL-17F and IL-17A reveals greater T-cell IL-17F induction and IL-23 regulation
Source: Front Immunol. 2025 Nov 14;16:1658325. doi: 10.3389/fimmu.2025.1658325 (PMC12660208; doi:10.3389/fimmu.2025.1658325)

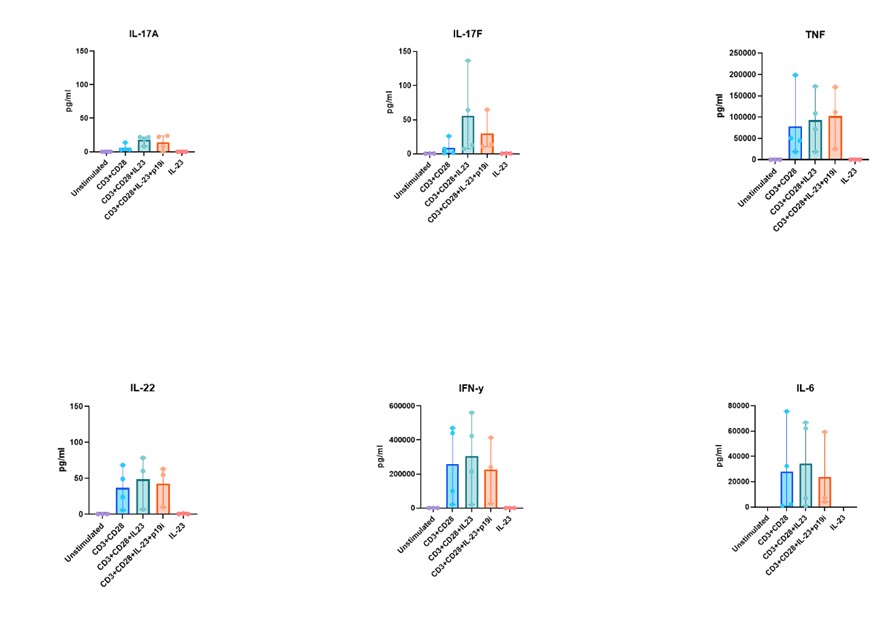

Supplement: Supplementary Figure 1 — IL-17F expression from T-cells is enhanced but not dependent on IL-23 Entheseal immune cells were stimulated for 72hrs using different T-cell stimulation conditions as well as IL-23 blockade with a p19 inhibitor. Cells seeded at 1x10^6^/ml were cultured with no stimulation, anti-CD3+anti-CD28 (100ng/ml), anti-CD3+anti-CD28 + IL-23(50ng/ml), anti-CD3+anti-CD28+IL-23+p19i (20ng/ml) or IL-23 alone (50ng/ml). Supernatant was collected after 72hrs and cytokine expression was assessed using a Legendplex assay for multi-analyte detection. N = 4 [file Image1.jpeg]

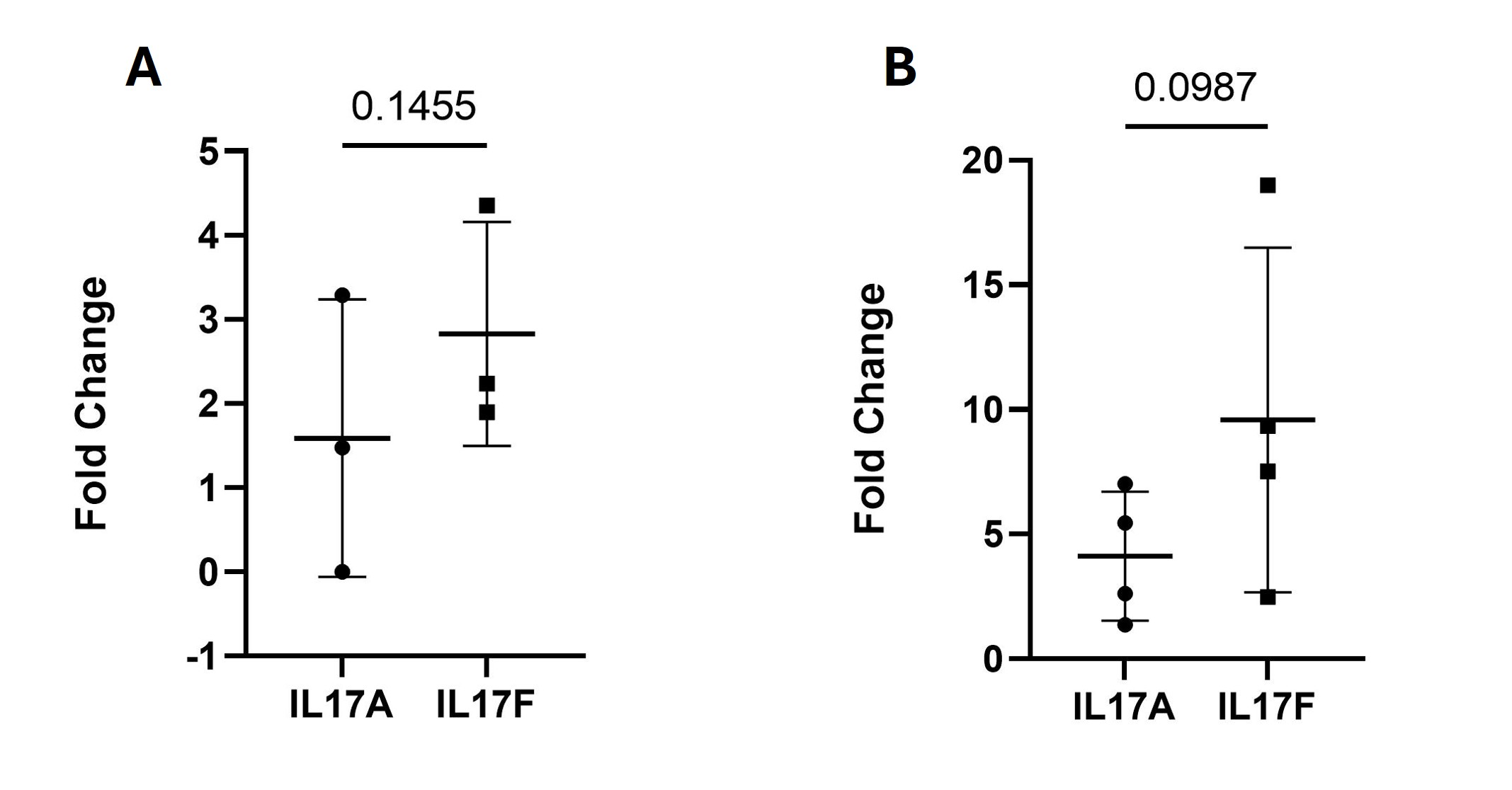

Supplement: Supplementary Figure 2 — Fold change of IL-17A and IL-17F expression with the addition of IL23 Fold change calculated between the CD3+CD28 stimulation and CD3+CD28+IL23 stimulation for IL-17A and IL-17F expression, (A) fold change is representative of the ELISA data n=3, (B) fold change representative of the Legendplex data n=4. Statistical analysis performed using paired t-test. [file Image2.jpeg]

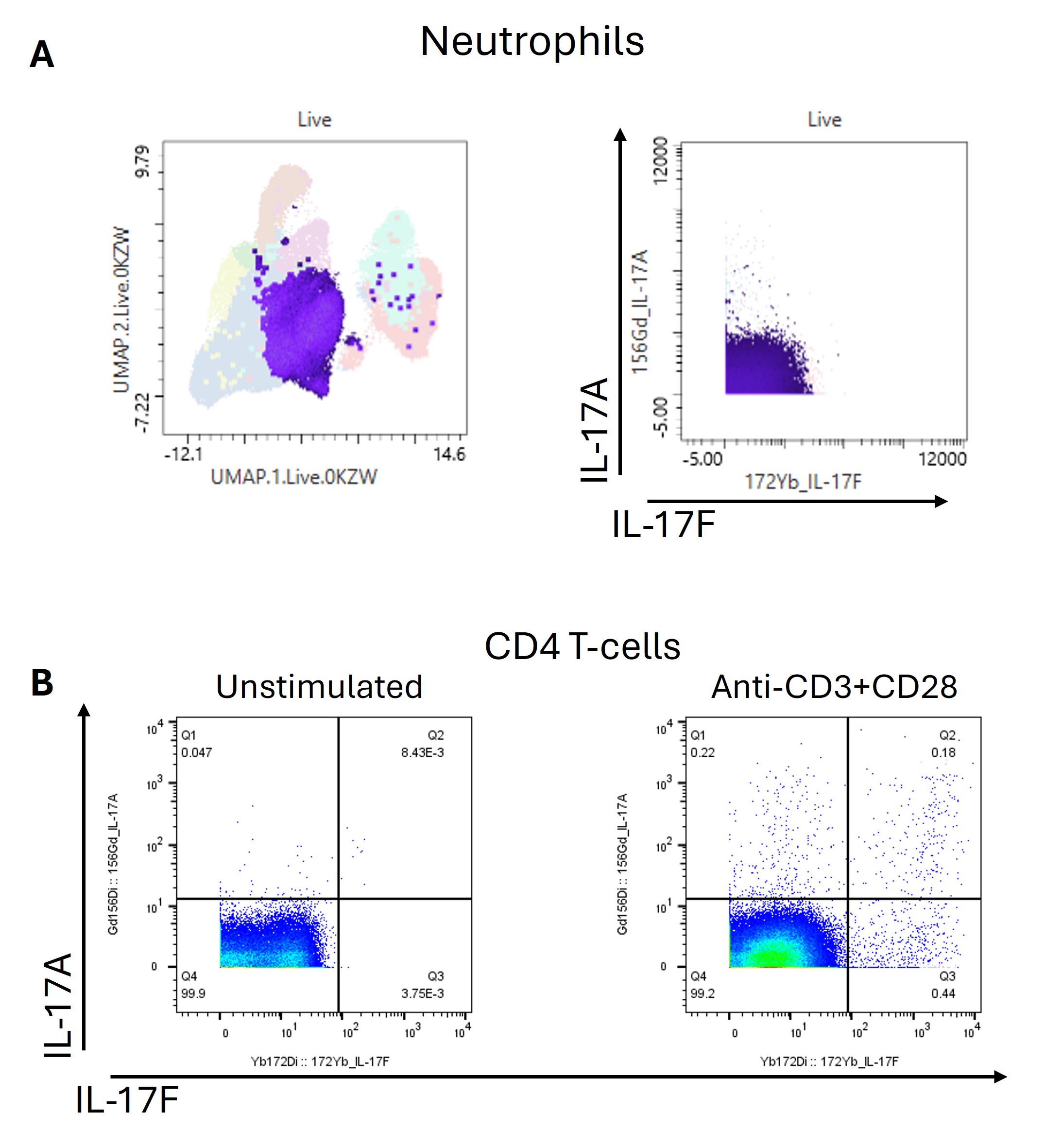

Supplement: Supplementary Figure — IL-17A & IL-17F expression from PEB neutrophils and CD4+ T-cells CyTOF analysis from PEB samples, cluster analysis performed on the stimulated (anti-CD3 & CD28) to isolate the neutrophil cluster and 2D plot to show IL-17A/IL-17F expression (A). 2D plots of the CD4+ T-cell populations from the unstimulated and stimulated samples showing IL-17A/IL-17F expression (B). [file Image3.jpeg]

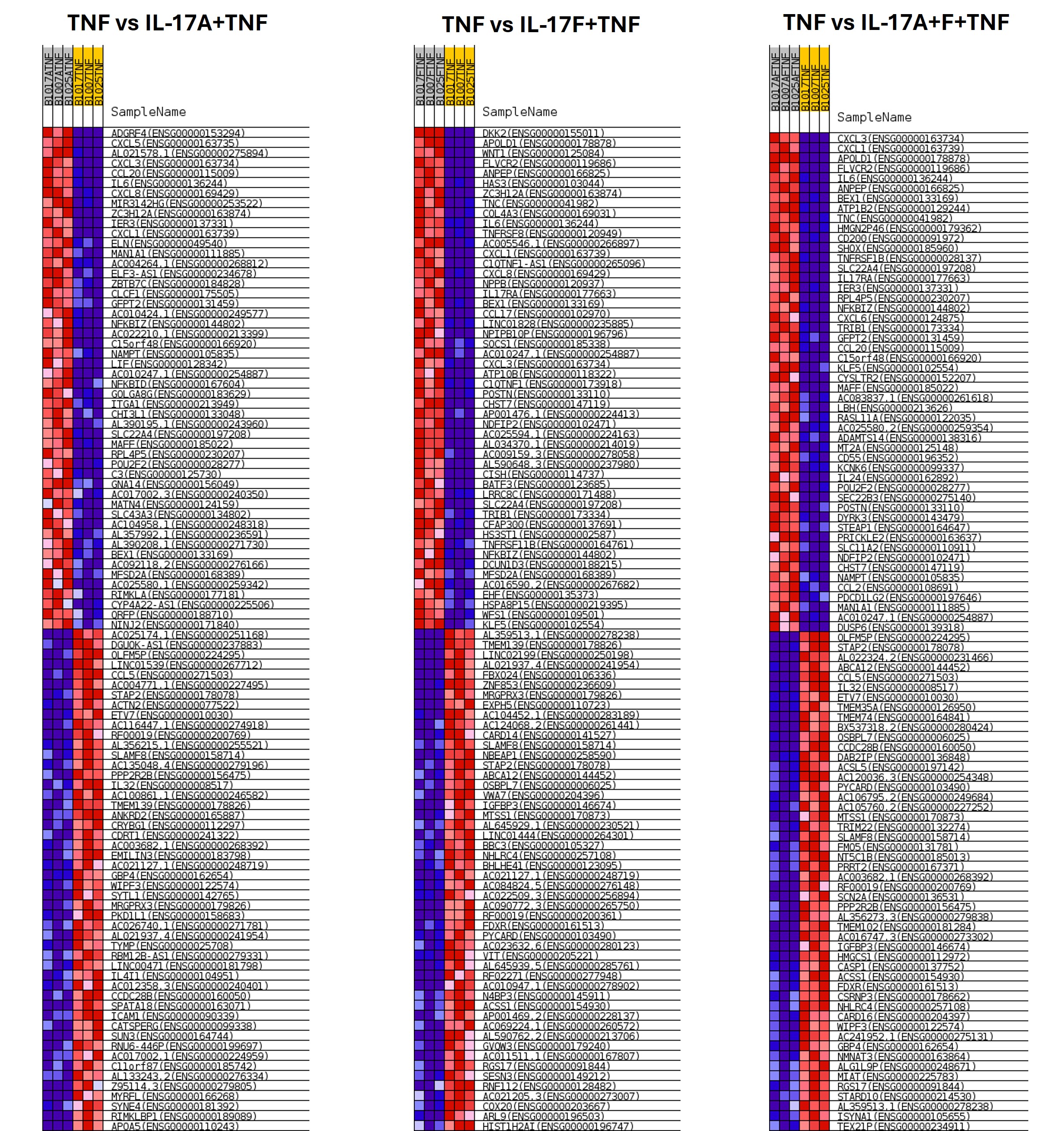

Supplement: Supplementary Figure 5 — Heatmaps of the top DEGS from MSCs stimulated with IL-17A, IL-17F and TNF Heatmaps of the up- and down-regulated differentially expressed genes (DEGs) from comparisons between TNF and conditions with the addition of IL-17A and IL-17F. Upregulated genes (red) and down-regulated genes (blue). [file Image4.jpeg]

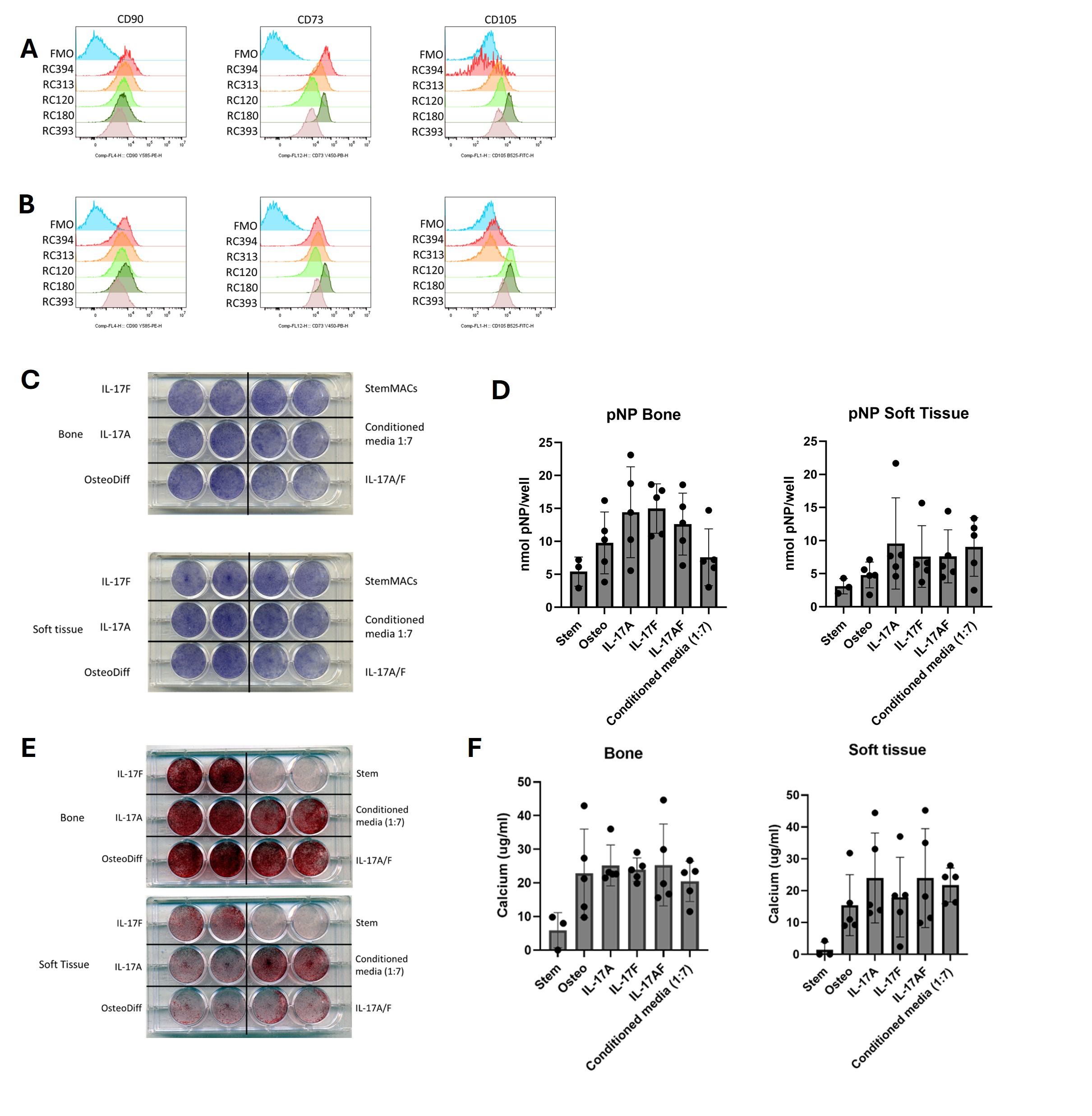

Supplement: Supplementary Figure 5 — Effects of IL-17A & IL-17F on osteogenesis. Osteogenic differentiations were set up in OsteoDiff media with the addition of recombinant IL-17A (50ng/ml), IL-17F (50ng/ml) and combined IL-17A/F (25/25ng/ml) and supernatant collected from activated T-cells after 72hr (1:7). StemMACs media was used as a control condition. At days 14 & 21 osteogenesis was assessed via staining methods, lysate quantification as well as RNA collection for gene expression. MSCs isolated from entheseal bone and soft tissue using collagenase digest were culture expanded and phenotyped for MSC markers CD90, CD73 & CD90 (A) -bone & (B) – soft tissue). Day 14 staining was performed for alkaline phosphatase with duplicate wells and levels of alkaline phosphatase quantified from lysates collected (C, E). Day 21 alizarin red staining performed and calcium quantification (D, F). MSCs isolated from entheseal bone and soft tissue were stimulated for 48hrs in the presence of IL-17A, IL-17F and TNF as well as in combination, MSC activation was measured by CCL20 expression by ELISA (G) N = 5 [file Image5.jpeg]
